# A 2-step strategy for detecting pleiotropic effects on multiple longitudinal traits

**DOI:** 10.3389/fgene.2014.00357

**Published:** 2014-10-20

**Authors:** Weiqiang Wang, Zeny Feng, Shelley B. Bull, Zuoheng Wang

**Affiliations:** ^1^Department of Mathematics and Statistics, University of GuelphGuelph, ON, Canada; ^2^Lunenfeld-Tanenbaum Research Institute of Mount Sinai Hospital, Prosserman Centre for Health ResearchToronto, ON, Canada; ^3^Dalla Lana School of Public Health, University of TorontoToronto, ON, Canada; ^4^Division of Biostatistics, Yale School of Public HealthNew Haven, CT, USA

**Keywords:** pleiotropic effect, genetic association, multiple traits, longitudinal data, mixed effects model, single nucleotide polymorphisms (SNPs)

## Abstract

Genetic pleiotropy refers to the situation in which a single gene influences multiple traits and so it is considered as a major factor that underlies genetic correlation among traits. To identify pleiotropy, an important focus in genome-wide association studies (GWAS) is on finding genetic variants that are simultaneously associated with multiple traits. On the other hand, longitudinal designs are often employed in many complex disease studies, such that, traits are measured repeatedly over time within the same subject. Performing genetic association analysis simultaneously on multiple longitudinal traits for detecting pleiotropic effects is interesting but challenging. In this paper, we propose a 2-step method for simultaneously testing the genetic association with multiple longitudinal traits. In the first step, a mixed effects model is used to analyze each longitudinal trait. We focus on estimation of the random effect that accounts for the subject-specific genetic contribution to the trait; fixed effects of other confounding covariates are also estimated. This first step enables separation of the genetic effect from other confounding effects for each subject and for each longitudinal trait. Then in the second step, we perform a simultaneous association test on multiple estimated random effects arising from multiple longitudinal traits. The proposed method can efficiently detect pleiotropic effects on multiple longitudinal traits and can flexibly handle traits of different data types such as quantitative, binary, or count data. We apply this method to analyze the 16th Genetic Analysis Workshop (GAW16) Framingham Heart Study (FHS) data. A simulation study is also conducted to validate this 2-step method and evaluate its performance.

## 1. Introduction

In genetics, the phenomenon that a single gene or locus influences more than one trait is known as pleiotropy. Genetic pleiotropy plays a crucial role in many complex diseases. One of the most well-known examples is the phenylketonuria (PKU) disease. The defect of a single gene supposed to code for enzyme phenylalanine hydroxyls results in multiple malfunctioned phenotypes such as mental retardation, eczema, and skin pigment defects. These phenotypes characterize the PKU disease (Lobo, 2008) and information on these phenotypes is often collected in PKU disease studies. For similar reasons, multiple disease related phenotypes are collected in many complex disease studies. For example, in coronary heart disease (CHD), phenotype information may include systolic blood pressure (SBP), low-density lipoprotein (LDL), high-density lipoprotein (HDL), triglycerides (TG), and other disease related measures. Combined analysis of these phenotypes may be more informative for etiologic study of the disease than analyzing each phenotype individually. If the objective is to identify genetic pleiotropic effects on multiple traits, the conventional approach is to perform an association test between a genetic variant and each trait individually and then look for consensus about whether the genetic variant is significantly associated with more than one trait. However, this approach inflates the family-wise error rate (FWER). The inflation becomes more severe as the number of traits increases. Usually, a multiple testing procedure is required to adjust the significance level of each individual test. On the other hand, when a genetic variant is associated with multiple traits, an individual test of each trait may ignore the extra information that is available from combining multiple traits in the analysis, thus leading to lower power. Therefore, a simultaneous genetic association test on multiple traits might be desirable to control the FWER and enhance the power of the analysis.

Several authors have proposed statistical methods for simultaneous association analysis of multiple traits. For example, Klei et al. (2008) proposed to test the association between an SNP and the principal component of heritability derived from multiple correlated traits. Ferreira and Purcell (2009) used canonical correlation analysis (CCA) to measure the association between an SNP and multiple traits. Zheng et al. (2010) proposed a non-parametric method based on the generalized Kendall's tau for the association between a marker and multiple traits. In joint analysis of the association across multiple phenotypic traits, Huang et al. (2010) used the multinomial regression model to model the distribution of the allele frequency of a given SNP among different phenotype outcomes. Huang et al. (2011) developed the PRIMe software tool to calculate the Pleiotropy Index (PI) over a region of SNPs where PI indicates the number of traits that have low *p*-values from the individual association test with each trait. However, these methods generally find SNPs that are consistently significant among multiple independent tests for each trait on the same dataset or via meta-analyses on different datasets. Hartley et al. (2012) proposed to use Bayesian network models to identify SNPs that are associated with one or more traits simultaneously. O'Reilly et al. (2012) proposed joint analysis of multiple phenotypes by regressing the genotype on multiple phenotypes. With a similar idea of regressing the genotype on multiple phenotypes, Feng (2014) proposed a generalized quasi-likelihood scoring approach for analyzing data from a sample of correlated subjects, such as data collected in family-based studies or isolated/founder population-based studies. Results from these studies generally confirm that simultaneous testing of multiple traits increases power compared to individual tests of each trait.

To effectively investigate the development of disease, longitudinal cohort studies are designed to obtain repeated measures of a variety of disease-related traits within an individual over time. For example, in CHD studies, repeated measurements of cardiovascular risk factors such as systolic blood pressure are taken over time as well as information on other covariates such alcohol consumption or smoking status. Despite the availability of multiple time point measurements for each subject, many genetic analyses only use one single time point measurement or an average over all time points for each subject. For example, Levy et al. (2000) regressed the mean of repeated blood pressure measurements of each subject on their age and body mass index in the first step. The residual for each subject from this regression model was used as a phenotype for the heritability and linkage analysis in the second step. However, this single time point measurement approach does not fully utilize the information provided in the data and thus can decrease the power of detecting the associated SNPs or underlying genes. For this reason, many methods have been developed to jointly analyze the genetic association with multiple time point measurements. One typical class of approaches is functional mapping, in which mathematical functions are used to establish the relationship between the underlying genes and the development or the progression of a complex trait. For example, Ma et al. (2002) proposed a logistic growth curve model for mapping quantitative trait loci (QTL) and estimating their effects. Wu and Lin (2006) provided an overview on the fundamental concepts of functional mapping and its application in QTL mapping and GWAS. Wu et al. (2007) proposed a semi-parametric functional mapping, a hybrid of a parametric function for earlier stages and a non-parametric function for late stages, to model the human immunodeficiency virus (HIV) progression and to study the genetic contributions to the HIV load trajectories. Das et al. (2011) proposed a so-called functional GWAS (*f*GWAS) based on nonparametric functions. In functional mapping, all measurements are utilized to capture the trajectories of the development or progression of a trait and thus a more powerful approach to unravel the genetic association with these trajectories.

On the other hand, mixed effects models have been a popular choice for modeling longitudinal data. Gauderman et al. (2003) summarized 13 contributions to the 13th Genetics Analysis Workshop in which methods for genetic analyses using longitudinal data are grouped into two basic approaches: the two-step approach and the joint modeling approach. In the two-step approach, repeated measurements of a phenotype is modeled by mixed effects models to reduce to one or two summary statistics for each subject in the first step and then, these subject-specific statistics will be used in the second step for the linkage or genetic analysis. In the joint modeling approach, a mixed effects model is used to jointly estimate genetic and longitudinal parameters. For example, genetic parameters may include additive polygenic and additive major gene effects. Longitudinal parameters may include shared environmental and random environmental effects. The joint modeling approach has also been proposed for genome-wide association mapping by Furlotte et al. (2012). Recently, rare variant association analysis has been an important direction in GWAS and most available methods for rare variant association are focusing on the effects of the weighted combination of variants. Wang et al. (2014) incorporated an optimally weighted combination of variants in a mixed effects model for detecting rare and common variants associated with a longitudinal trait. Results from these studies confirm an improved power when all time points are jointly analyzed. However, currently available methods focus on the analysis of one longitudinal trait at a time. A new method that can effectively and simultaneously analyze multiple longitudinal traits, particularly for identifying genetic pleiotropic association, is desirable. Further more, a method that can flexibly and simultaneously handle traits of different data types such as quantitative, binary, or count data, would be attractive.

In this paper, we propose a 2-step strategy for analyzing the association of a genetic variant with multiple longitudinal traits. In the first step, a mixed effects model is used to analyze the repeated measurements for each trait individually. The subject-specific random effect is used to extract the component of variation that includes genetic factors contributing to the trait for each individual subject. Throughout this paper, we refer to this random effect as the subject-specific effect. The fixed effects account for observed confounding factors such as environmental factors and some time-dependent variables. In the second step, we treat the estimated subject-specific effect as a phenotype. We propose to regress the genotype of a genetic variant on all estimated subject-specific effects for the traits and test the association between the genetic variant and these subject-specific effects simultaneously through the score test or the likelihood ratio test.

The remainder of our paper is organized as follows. In Section 2, we describe the proposed method and the details of the simulation study. We also apply our method to analyze data from the 16th Genetic Analysis Workshop (GAW16) Framingham Heart Study. Results from simulation study and data analysis application are presented in Section 3. Discussion and possible future study follow in Section 4.

## 2. Materials and methods

This section consists of three subsections. The first subsection describes our proposed 2-step method. A simulation study is present in the second subsection. In the third subsection, we apply our method to analyze the data from GAW16 Framingham Heart Study.

### 2.1. Statistical method

In this subsection, we begin by defining a generalized linear mixed effect model for each of the multiple longitudinal traits of interest. Each model can include time-dependent or time-independent covariates together with a random effect for subject-specific effect. This constitutes Step 1 of the proposed method. Then, we introduce a binomial regression model that treats the genotype as the response variable and includes multiple subject-specific genetic effects obtained in Step 1 for each longitudinal trait as the explanatory covariates. This constitutes Step 2 of the proposed method.

#### 2.1.1. Step 1: generalized linear mixed models (GLMMs) for longitudinal traits

In longitudinal study designs, repeated measurements of phenotypic traits and covariates are taken for each subject over time. GLMMs are useful for modeling phenotypes of different data types, such as quantitative, binary, and count data. Suppose we have a sample of *n* independent subjects in our study. For each subject *i*, *i* = 1, 2, …, *n*, we collect repeated measurements on *J* different traits. Let ***X**_ij_* = (*X*_*ij*1_, …, *X_ijt_*, …, *X_ijT_ij__*)′ be a vector that represents the *T_ij_* measurements of the *j*th trait for subject *i* and so *X_ijt_* is the *t*th measurement of the *j*th trait for subject *i*. A general form of a GLMM can be expressed as

(1) $$g_{j}(\mu_{ijt})=Z_{ijt}^{T}\alpha_{j}+\gamma_{ij},$$

where *g_j_*(.) is the link function for the *j*th trait, **Z**_*ijt*_ is a vector of covariates associated with the *j*th trait for the *i*th subject at time *t*, **α**_j_ is the vector of fixed effects for covariates **Z**_*ijt*_, γ_*ij*_ is the random effect representing the *i*th subject-specific effect on the *j*th trait, and μ_*ijt*_ is the conditional mean of *X_ijt_* given **Z**^*T*^_*ijt*_ and γ_*ij*_. Here, the associated covariates can be time-dependent or time-independent. Examples of time-dependent covariates include treatment status and age at each measurement time. Time-independent covariates such as sex are treated as constants over time. We allow different sets of covariates to be considered for different traits and the number of measurements *T* can be different for each subject as well. The subject-specific effect γ_*ij*_ can be interpreted as the influence of subject *i* on his/her repeated measurements on the *j*th trait and it typically includes genetic effects on the trait. So, the γ_*ij*_'s can capture the effects of unobserved major genes and polygenes; the latter refers to the combined effects of a large number of genetic variants that each make a small contribution to trait variation. For each trait, say the *j*th trait, we assume the γ_*ij*_'s follow a normal distribution with a mean of 0 and a trait specific variance σ^2^_γ_*j*__.

For a quantitative trait, a linear mixed effects model can be used, for example

$$X_{ijt}=Z_{ijt}^{T}\alpha_{j}+\gamma_{ij}+\epsilon_{ijt},$$

where random error ϵ_*ijt*_ is assumed to follow a *N* (0, σ^2^_ϵ_*j*__) distribution. Then, *g_j_*(.) is an identity link with *g_j_(μ_ijt_) = μ_ijt_*. For a binary trait, a logistic link can be used with $g_{j}(\mu_{ijt})=\log\left(\frac{\mu_{ijt}}{1-\mu_{ijt}}\right)$. The GLMMs can be fitted in R by the “lme4” package (Bates et al., 2013). The estimated $\hat{\gamma }$_*ij*_'s will be treated as phenotypic traits for the association analysis in Step 2. The fixed effects associated with confounding factors can be estimated using the “lme4” package as well.

For different longitudinal data types, we interpret the associated subject-specific effect accordingly. When a longitudinal trait is binary, for example if the *j*th trait being considered is hypertension status, γ_*ij*_ can be interpreted as the underlying genetic risk factors of subject *i* that affect the log-odds for the risk of hypertension. When the *j*th longitudinal trait of interest is the daily seizure count of an epilepsy patient, γ_*ij*_ can be interpreted as the underlying genetic risk of subject *i* that affects the log of the daily seizure rate.

#### 2.1.2. Step 2: genetic association study with multiple longitudinal traits

Single nucleotide polymorphisms (SNPs) are the most common genetic variants in human and animal genomes. Because association studies are nearly all conducted using SNP data, our method will focus on applications to SNP association studies. Most SNPs are biallelic so, without loss of generality, for each SNP, we label the two alleles as “0” or “1”; the possible genotypes for this SNP are 0, 1, or 2 for the count of copies of the less frequent allele 1. Let **Y** = (*Y*_1_, *Y*_2_, …, *Y_n_*) ′ be a vector of observed proportions of allele 1 of a given SNP for *n* unrelated subjects. So, *Y_i_* takes values of 0, $\frac{1}{2}$, or 1. Let ***p*** = (*p*_1_,*p*_2_, …,*p_n_*)′ be a vector of the expected frequency of allele 1 in this SNP for *n* subjects and 0 < *p_i_* < 1 for all *i*. Then, under the Hardy-Weinberg equilibrium, 2*Y_i_* follows a binomial(2, *p_i_*) distribution and the log-likelihood function over *n* unrelated subjects has the form

$$l(p)=\sum_{i=1}^{n}\left\{2Y_{i}\log\left(\frac{p_{i}}{1-p_{i}}\right)+2\log(1-p_{i})\right\}.$$

Let **γ** be an *n* × (*J* + 1) design matrix of the form

$$\gamma =\left(\begin{array}{cccc} 1 & \gamma_{11} & \cdots & \gamma_{1J}\\ 1 & \gamma_{21} & \cdots & \gamma_{2J}\\ \vdots & \vdots & \ddots & \vdots \\ 1 & \gamma_{n1} & \cdots & \gamma_{nJ} \end{array}\right).$$

where the (*j*+1)th column represents the subject-specific effects corresponding to the *j*th longitudinal trait for all subjects and the *i*th row, **γ***_i_*, contains a 1 for the intercept and the *J* subject-specific effects for subject *i*. With a logistic link,

$$p_{i}=E(Y_{i}|\gamma_{i})=\frac{\exp\{\gamma_{i}^{T}\beta \}}{1+\exp\{\gamma_{i}^{T}\beta \}}$$

If the SNP being tested is associated with a longitudinal trait, it should be associated with its corresponding subject-specific effect which includes the contribution of genetic factors to the variation of the trait. On the other hand, if the SNP is not associated with any one of the *J* longitudinal traits, it would not be associated with the corresponding subject-specific effect and all coefficients β_1_, …, β_*J*_ should be 0. So, a simultaneous association test between the SNP and the *J* longitudinal traits can be formulated as an overall hypothesis test that

$$\begin{array}{l} H_{0}:\beta_{1}=\beta_{2}=\cdots =\beta_{J}=0\text{ against}\\ H_{a}:\text{ at least one }\beta_{j}\neq 0,j=1,\;2,\ldots ,\;J, \end{array}$$

Here, we can use either Rao's score test statistic (Rao, 1948) or the likelihood ratio test (LRT) statistic to test the hypothesis.

Under *H*_0_ that β's are all 0, $p_{i}=\frac{\exp\{\beta_{0}\}}{1+\exp\{\beta_{0}\}}=p$ is a constant for all subjects. The maximum likelihood estimator (MLE) of *p* under *H*_0_, denoted by $\tilde{p}$, is given by $\bar{Y}=\frac{\sum_{i=1}^{n}Y_{i}}{n}$, and thus ${\tilde{\beta }}_{0}=\log\left\{\frac{\tilde{p}}{1-\tilde{p}}\right\}$. The Rao's score test statistic under *H*_0_, denoted by *W_s_*, is given by

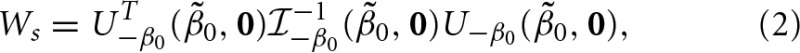



where *U* ($\tilde{\beta }$_0_,**0**) is a vector of the score functions computed under the null hypothesis and β_0_ = $\tilde{\beta }$_0_. The subscript of *U*_−β_0__($\tilde{\beta }$_0_,**0**) indicates the removal of the first term (i.e., the intercept term) from *U* ($\tilde{\beta }$_0_,**0**). 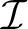
($\tilde{\beta }$_0_,**0**) is the observed information matrix of **β** computed under the null hypothesis and β_0_ = $\tilde{\beta }$_0_. The subscript of 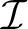
^−1^_−β_0__($\tilde{\beta }$_0_,**0**) indicates the removal of the first row and the first columns corresponding to β_0_ from 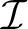
^−1^($\tilde{\beta }$_0_,**0**). Based on Equation (2), we derive an explicit form of score statistics as follows,

(3) $$W_{s}=\frac{2}{\tilde{p}(1-\tilde{p})}{\left(Y-\tilde{p}1\right)}^{T}\gamma_{-1}{\left(\gamma^{T}\gamma \right)}_{-1}^{-1}\gamma_{-1}^{T}(Y-\tilde{p}1),$$

where **γ**_−1_ indicates the removal of the first column of design matrix **γ**, (**γ**^*T*^**γ**)^−1^_−1_ represents the removal of the first row and the first column of (**γ**^*T*^**γ**)^−1^, and **1** is a vector of 1's. Under *H*_0_, *W_s_* follows an asymptotic χ^2^_*J*_ distribution with *J* being the number of traits to be tested.

Straightforwardly, the LRT statistic, Λ = −2{*l*($\hat{\beta }$) − *l*($\tilde{\beta }$) with $\hat{\beta }$ being the unrestricted MLEs of **β** and $\tilde{\beta }$ being the restricted MLEs of **β** under the null hypothesis that β_1_ = β_2_ = ··· = β_*K*_ = 0, takes the form

(4) $$\Lambda =-2\sum_{i=1}^{n}\left\{2Y_{i}\log\left(\frac{{\hat{p}}_{i}}{\tilde{p}}\right)+(2-2Y_{i})\log\left(\frac{1-{\hat{p}}_{i}}{1-\tilde{p}}\right)\right\}$$

where ${\hat{p}}_{i}=\frac{\exp\{\gamma_{i}^{T}\hat{\beta }\}}{1+\exp\{\gamma_{i}^{T}\hat{\beta }\}}$. Under *H*_0_, Λ follows an asymptotic χ^2^*_J_* distribution with *J* being the number of traits to be tested. Note that these subject-specific effects γ_*ij*_'s are not observable. To compute the *W_s_* and Λ statistics using Equations (3) and (4), we plug in the estimated subject-specific effects $\hat{\gamma }$_*ij*_ to replace the γ_*ij*_'s.

### 2.2. Simulation studies

To assess the performance of the proposed method, we conducted simulation studies evaluating the type I error rate and the power of the association tests. Our simulation studies accommodate two different designs. In both studies, we consider two quantitative traits and one binary trait. These three traits can be affected by three SNPs, denoted by *G*_1_, *G*_2_, and *G*_3_, at different levels. In the first study, each SNP affects all three traits. In the second study, each SNP can affect a different number of traits, as specified in Table 1.

**Table 1:** **SNP effects on three traits for simulation study 1 and 2**.

**Study 1**				**Study 2**			
**SNP**	**Trait**			**SNP**	**Trait**		
	***X*_1_**	***X*_2_**	***X*_3_**		***X*_1_**	***X*_2_**	***X*_3_**
*G*_1_	Yes	Yes	Yes	*G*_1_	Yes	Yes	Yes
*G*_2_	Yes	Yes	Yes	*G*_2_	Yes	No	Yes
*G*_3_	Yes	Yes	Yes	*G*_3_	No	Yes	No

In many situations, trait-causal SNPs may not be genotyped but instead, SNPs that are close to or in linkage disequilibrium (LD)/associated with these causal SNPs are available in the study. So, in our simulation study, we consider testing on both the causal SNPs and SNPs that are associated with these causal SNPs. Suppose we generate a sample of *n* independent subjects. For each subject *i*, we generate genotypes of three independent trait-causal SNPs, *G*_1_, *G*_2_, and *G*_3_, and genotypes of three SNPs, *M*_1_, *M*_2_, and *M*_3_, that are in LD with *G*_1_, *G*_2_, and *G*_3_ respectively. To generate SNP genotypes, we generate a haplotype for each pair of associated SNPs. Let **H**_*r*_ = (*H_G_r__, H_M_r__*) be the haplotype for SNPs *G_r_* and *M_r_* for *r* = 1, 2, 3. The haplotype **H***_r_* is generated from a bivariate Bernoulli distribution with mean vector **π**_*r*_ = (π_*G_r_*_, π_*M_r_*_)^′^ and covariance matrix

(5) $$\Sigma_{r}=\;\left(\begin{array}{cc} \sigma_{G_{r}}^{2} & \sigma_{G_{r},M_{r}}^{2}\\ \sigma_{G_{r},M_{r}}^{2} & \sigma_{M_{r}}^{2} \end{array}\right),$$

where σ^2^*_G_r__* = π*_G_r__*(1 − π*_G_r__*), σ^2^_*M_r_*_ = π_*M_r_*_(1 − π*_M_r__*), and σ^2^_*G_r_,M_r_*_ = ρ_*r*_σ_*G_r_*_σ_*M_r_*_ with ρ_*r*_ being the correlation between the SNPs *G_r_* and *M_r_*. **π**_*r*_ is a vector of frequencies of allele 1 for SNPs *G_r_* and *M_r_*. We set **π**_1_ = (0.1, 0.2)′, **π**_2_ = (0.15, 0.4)′, and **π**_3_ = (0.2, 0.3)′. We then specify the correlations with ρ_1_ = 0.95, ρ_2_ = 0.9, and ρ_3_ = 0.85. A pair of **H**_*r*_ are generated to make up the genotypes of *G_r_* and *M_r_*. We also simulate an independent SNP *M* for the purpose of Type I error rate assessment. The genotype of SNP *M* is simulated from binomial(2, 0.2).

Then we generate two general covariates *Z*_*it*1_ and *Z*_*it*2_ for subject *i* at the *t*th measurement. The covariates can be time-varying or time-invarying. When the covariate is time-invarying, it will be a constant with respect to *t*. Here, we generate time-varying covariates for both *Z*_*it*1_ and *Z*_*it*2_. We let the total number of measurements be *T* = 5 for each subject. We let *Z*_*it*1_ be a binary covariate generated from Bernoulli(0.3) and let *Z*_*it*2_ be a quantitative covariate generated from *N* (μ, σ^2^). We let μ = 40 and σ = 7 to mimic the age distribution of patients, which is a typical time-varying covariate in longitudinal data. Thus, the *Z*_*it*2_'s are sorted in ascending order such that *Z*_*i*12_ < ··· < *Z*_*it*2_ < ··· < *Z*_*iT*2_.

Given the generated covariates and the genotypes of causal SNPs, we generate measurements of each trait for each subject by first computing the linear predictors given by

$$\begin{array}{l} \eta_{ijt}=g(\mu_{ijt})=\alpha_{j0}+\alpha_{j1}Z_{it1}+\alpha_{j2}Z_{it2}+b_{j1}G_{i1}+b_{j2}G_{i2}\\ \;+\;b_{j3}G_{i3}\text{,} \end{array}$$

for *i* = 1, …, *n, j* = 1, 2, 3, and *t* = 1, …, 5. The two quantitative traits, *X*_*i*1*t*_ and *X*_*i*2*t*_, are generated from *N* (μ_*i*1*t*_, 1) and *N* (μ_*i*2*t*_, 1) with identity links η_*i*1*t*_ = μ_*i*1*t*_ and η_*i*2*t*_ = μ_*i*2*t*_, respectively. The binary trait, *X*_*i*3*t*_, is generated from Bernoulli(μ_*i*3*t*_), where $\mu_{i3t}=\frac{\exp\{\eta_{i3t}\}}{1+\exp\{\eta_{i3t}\}}$.

In simulation study 1, we set **α**_1_ = (α_10_, α_11_, α_12_)^*T*^ = (0, 0.3, 0.5)^*T*^ and **b**_1_ = (**b**_11_, **b**_12_, **b**_13_)^*T*^ = (0.25, 0.2, 0.2)^*T*^ for the first quantitative trait *X*_*i1t*_. For the second quantitative trait *X*_*i*2*t*_, we set **α**_2_ = (0, 0.2, −0.3)^*T*^ and **b**_2_ = (0.25, 0.25, 0.15)^*T*^. For the binary trait *X_i3t_*, we set **α**_3_ = (−0.3, −0.6, 0.35)^*T*^ and **b**_3_ = (0.45, 0.4, 0.3)^*T*^ such that the simulated sample consists of about 40% cases and 60% controls. In simulation study 2, the fixed effects **α**_*j*_'s that are associated with the covariates *Z*'s in study 2 remain the same as in study 1. However, we set **b**_1_ = (0.25, 0.22, 0)^*T*^, **b**_2_ = (0.2, 0, 0.15)^*T*^, and **b**_3_ = (0.45, 0.43, 0)^*T*^, such that, SNP *G*_1_ affects all three traits, SNP *G*_2_ affects two traits, and SNP *G*_3_ affects one trait only.

For each simulation study, we generate samples of size *n* = 100, 200, and 300 and, for each specified sample size, we simulate 1000 data sets. For each data set, we first fit the GLMM to obtain an estimate of γ_*ij*_ for each trait and each subject. In the GLMMs, both covariates, *Z*_*it*1_ and *Z*_*it*2_, are included. For each SNP, we then perform a simultaneous test on all three estimated subject-specific effects; $\hat{\gamma }$_1_, $\hat{\gamma }$_2_ and $\hat{\gamma }$_3_, where each $\hat{\gamma }$*j* = ($\hat{\gamma }$_1*j*_, …, $\hat{\gamma }$_*nj*_)^*T*^ is treated as a phenotype. Because we simultaneously test on three phenotypes, both *W_s_* and Λ test statistics follow a χ^2^_3_ distribution asymptotically under the null hypothesis. We reject the null hypothesis if the test statistic is greater than the (1 − α_*F*_)th quantile of the χ^2^_3_ distribution. We let α_*F*_ = 0.05, 0.01, and 0.001. We also perform individual association tests between each SNP and each subject-specific effect for each trait. We reject the null hypothesis if the test statistic computed for only one estimated subjected-specific effect has a value greater than the (1 − α)th quantile of χ^2^_1_ distribution. Here, α is given by α_*F*_ = 1 − (1 − α)^3^ and α_*F*_ is the family-wise error rate (FWER) controlling at 0.05, 0.01, and 0.001 levels.

We also consider different sets of covariates and fixed effects in our simulation studies. The results demonstrate similar patterns in terms of power and empirical type I error rates of association tests when different scenarios for fixed effects are considered. Please see the Supplementary Material for other simulation models and their corresponding results.

### 2.3. Application to GAW16 framingham heart study data set

Our proposed method is used to analyze the 16th Genetic Analysis Workshop (GAW16) Framingham Heart Study (FHS) data. The GAW16 FHS data are drawn from the FHS under the direction of the National Heart, Lung, and Blood Institute. The FHS aims to identify risk factors that contribute to cardiovascular disease (CVD). Data from families from the town of Framingham, Massachusetts (USA) were collected between 1948 and 2005 to a maximum of three generations. The FHS consists of three cohorts. The first cohort consists of the original participants in the first generation. The second cohort is the offspring recruited from children of the original participants and the spouses of these children. The third cohort consists of the third generation, which are the offsprings of the second generation. Most participants have repeated measurements on phenotypic traits from four examinations. Among the three cohorts, the offspring cohort possesses the most complete genotype data and phenotype information from the four exams.

Our analysis in this paper focuses on the offspring cohort. From this cohort, we select a subset of 1817 unrelated children using an algorithm as described in the R function“pedigree.unrelated” in the package “kinship2” (Therneau et al., 2012). We further remove two people from this subset for the analysis because they missed more than two exams. We consider four CVD-related longitudinal traits: SBP, LDL, HDL, and TD. We also include both time-invariant and time-variant covariates as potential confounding factors in our analysis. Time-invariant covariates include sex and type II diabetes diagnosed during the study period (diabetes = 0 for no, 1 for yes). Time-variant covariates include age, body mass index (bmi), smoking status (smk = 0 for never, 1 for former smoker, 2 for current smoker), number of cigarette smoked per day (cigs), number of alcoholic beveages consumed in ounce per week (alc), treatments for hypertension (htnrx = 0 for no, 1 for yes), and treatment for cholesterol (cholrx1 = 0 for no, 1 for yes) measured at each exam. All subjects included in the analysis have at least three repeated measurements consistently taken on all traits and time-variant covariates. All subjects were genotyped using the Affymetrix GeneChip Human Mapping 500 k array set. In total, we include 479,207 SNPs on 22 autosomes in our analysis. When testing each SNP, subjects with missing genotypes are excluded from the analysis at that SNP.

In Step 1, we first take log-transformations of SBP, HDL, and TG to adjust the skewness of their distributions. The R function “bfFixefLMER_F.fnc” in the “LMERConvenienceFunctions” package (Tremblay and Ransijn, 2013) is used to select covariates to be included in the linear mixed effects model. We then fit a linear mixed effects model to each longitudinal trait to obtain an estimated subject-specific effect for each trait and each individual. Then in Step 2, we simultaneously test the association between each SNP and all four estimated subject-specific effects corresponding to the four traits. We also perform individual association tests between each SNP and each estimated subject-specific effect for each trait.

## 3. Results

### 3.1. Simulation study results

In Table 2, the mean and standard error of fixed effects estimates, $\hat{\alpha }$'s, over 1000 simulations are reported and they are compared with the true values of each fixed effect used to generate the three longitudinal traits. The results of both simulation studies show that the GLMMs generally give unbiased estimates for the fixed effect parameters with small standard errors.

**Table 2:** **Mean and standard error of fixed effects estimates using GLMMs and based on over 1000 simulations for sample size *n* = 100**.

**Traits**	**Fixed effect**	**Study 1**		**Study 2**	
		**Estimate**	***SE***	**Estimate**	***SE***
1	α_11_ = 0.3	0.299	0.057	0.302	0.056
	α_12_ = 0.5	0.499	0.003	0.499	0.003
2	α_21_ = 0.2	0.199	0.057	0.201	0.056
	α_22_ = −0.3	−0.299	0.003	−0.300	0.003
3	α_31_ = −0.6	−0.601	0.147	−0.611	0.147
	α_32_ = 0.35	0.354	0.016	0.353	0.016

#### 3.1.1. Type I error rate assessment

For SNP *M* that is not associated with any trait in either study 1 or study 2, the empirical null rejection rates are reported in Table 3 for different sample sizes. We also combine the results from both studies (indicated as “Study 1+2”) so that we have 2000 simulation replicates to assess the type I error rate. For simultaneous tests, the empirical null rejection rates are very close to their corresponding nominal levels, indicating that the method controls the type I error properly. For individual tests, the null rejection rates are almost identical between the score test and the LRT, so we only report the results based on the LRT. The results indicate that the null rejection rates for each individual trait are very close to their corresponding nominal level. The union of individual null rejection rates reports the overall type I error rates among the three individual tests. These overall null type I error rates are very close to the theoretical FWERs α_*F*_'s.

**Table 3:** **Type I error rate assessment based on 1000 simulations in each study**.

**Sample size**	**α_*F*_**	**Individual tests**				**Simultaneous test**	
		**1**	**2**	**3**	**Union**	**Score**	**LRT**
***N* = 100**							
	0.05	0.015	0.016	0.014	0.04	0.045	0.047
Study 1	0.01	0.003	0.003	0.003	0.009	0.013	0.018
	0.001	0	0.001	0	0.001	0.002	0.002
	0.05	0.01	0.015	0.013	0.038	0.038	0.042
Study 2	0.01	0.004	0.001	0.003	0.008	0.01	0.008
	0.001	0.001	0	0	0.001	0.001	0.003
	0.05	0.0125	0.0155	0.0135	0.039	0.0415	0.0445
Study 1 + 2	0.01	0.0035	0.002	0.003	0.0085	0.0115	0.013
	0.001	0.0005	0.0005	0	0.001	0.0015	0.0025
***N* = 200**							
	0.05	0.014	0.017	0.014	0.042	0.042	0.044
Study 1	0.01	0.001	0.003	0.003	0.007	0.014	0.014
	0.001	0	0	0	0	0	0
	0.05	0.014	0.02	0.02	0.052	0.048	0.05
Study 2	0.01	0.002	0.003	0	0.005	0.007	0.007
	0.001	0	0	0	0	0.001	0.003
	0.05	0.014	0.0185	0.017	0.047	0.045	0.047
Study 1 + 2	0.01	0.0015	0.003	0.0015	0.006	0.0105	0.0105
	0.001	0	0	0	0	0.0005	0.0015
***N* = 300**							
	0.05	0.02	0.015	0.015	0.048	0.054	0.054
Study 1	0.01	0.004	0.003	0.002	0.009	0.013	0.013
	0.001	0	0.001	0	0.001	0.002	0.002
	0.05	0.015	0.017	0.025	0.057	0.051	0.055
Study 2	0.01	0.003	0.002	0.003	0.008	0.008	0.009
	0.001	0.001	0	0	0.001	0.001	0.001
	0.05	0.0175	0.016	0.02	0.0525	0.0525	0.0545
Study 1 + 2	0.01	0.0035	0.0025	0.0025	0.0085	0.0105	0.011
	0.001	0.0005	0.0005	0	0.001	0.0015	0.0015

Individual test results are based on the LRT.

For α_F_ = 0.05, 0.01^*^, 0.001^**^, α = 0.0167, 0.0033^*^, 0.00033^**^, respectively.

#### 3.1.2. Power assessment

The empirical power for each causal SNP and associated SNP are reported in Tables 4–6 for different sample sizes. In individual trait tests, we report only the results based on the LRT because the differences between the score test and the LRT are negligible. In study 1, all causal SNPs (i.e., *G*_1_, *G*_2_, *G*_3_) have genetic effects on all three longitudinal traits. When testing the association between each causal SNP and all three subject-specific effects simultaneously, the power is consistently higher than the power obtained from the union of three individual tests. When testing SNPs *M*_1_, *M*_2_, and *M*_3_ that are in LD with the causal SNPs, the simultaneous tests are also consistently more powerful than the union of individual tests. Certainly, the power is diluted in comparison with the tests on the trait-causal SNPs. In study 2, SNP *G*_1_ affects all three longitudinal traits, SNP *G*_2_ affects the first and the third longitudinal traits (*X*_*i1t*_ and *X_i3t_*), and SNP *G*_3_ affects one longitudinal trait only (*X_i2t_*). We observe that when the SNPs are associated with more than one trait, the simultaneous test is consistently more powerful than the union of individual tests for different sample sizes. The power gain is more obvious when the SNP is associated with more traits. When the SNP is associated with one trait, the power of the simultaneous trait test is similar to the individual trait test. Again, when testing on SNPs *M*_1_, *M*_2_, and *M*_3_ that are in LD with causal SNPs, the power is generally diluted. However, similar patterns to those obtained from tests of causal SNPs are observed. Note that in Tables 4–6, values in parentheses represent empirical type I error rates. For example, *G*_2_ in study 2 is not associated with the second longitudinal trait, so, its empirical rejection rate corresponds to the type I error rate.

**Table 4:** **Power comparisons for sample size 100 based on 1000 replications**.

	**α_*F*_**	**Individual tests**				**Simultaneous test**	
		**1**	**2**	**3**	**Union**	**Score**	**LRT**
**STUDY 1**							
	0.05	0.472	0.436	0.149	0.697	0.789	**0.796**
*G*_1_	0.01	0.258	0.224	0.06	0.423	0.565	**0.571**
	0.001	0.073	0.068	0.011	0.14	0.29	**0.304**
	0.05	0.436	0.666	0.205	0.824	0.886	**0.888**
*G*_2_	0.01	0.228	0.418	0.087	0.562	0.69	**0.701**
	0.001	0.055	0.17	0.013	0.214	0.406	**0.429**
	0.05	0.539	0.297	0.167	0.703	0.746	**0.755**
*G*_3_	0.01	0.285	0.118	0.056	0.399	0.52	**0.531**
	0.001	0.088	0.025	0.011	0.118	0.224	**0.247**
	0.05	0.257	0.233	0.092	0.446	0.485	**0.489**
*M*_1_	0.01	0.119	0.094	0.029	0.203	0.269	**0.273**
	0.001	0.023	0.022	0.005	0.047	0.103	**0.107**
	0.05	0.13	0.238	0.076	0.367	0.399	**0.405**
*M*_2_	0.01	0.055	0.107	0.024	0.169	0.185	**0.194**
	0.001	0.009	0.017	0.001	0.027	0.055	**0.062**
	0.05	0.307	0.183	0.1	0.462	0.501	**0.511**
*M*_3_	0.01	0.138	0.061	0.027	0.208	0.267	**0.285**
	0.001	0.032	0.008	0.002	0.042	0.09	**0.102**
**STUDY 2**							
	0.05	0.498	0.316	0.195	0.7	0.82	**0.826**
*G*_1_	0.01	0.265	0.134	0.064	0.386	0.612	**0.633**
	0.001	0.074	0.026	0.013	0.107	0.319	**0.342**
	0.05	0.528	(0.01)	0.253	0.632	0.723	**0.729**
*G*_2_	0.01	0.317	(0)	0.118	0.39	0.479	**0.491**
	0.001	0.099	(0)	0.032	0.125	0.216	**0.236**
	0.05	(0.007)	0.359	(0.014)	0.359	0.395	**0.405**
*G*_3_	0.01	(0)	0.18	(0.003)	0.18	0.172	**0.186**
	0.001	(0)	0.041	(0)	0.041	0.041	**0.058**
	0.05	0.281	0.192	0.114	0.46	0.548	**0.557**
*M*_1_	0.01	0.134	0.077	0.039	0.23	0.327	**0.343**
	0.001	0.031	0.016	0.004	0.051	0.131	**0.148**
	0.05	0.195	(0.01)	0.092	0.261	0.301	**0.306**
*M*_2_	0.01	0.079	(0.002)	0.024	0.099	0.119	**0.129**
	0.001	0.011	(0)	0.004	0.015	0.023	**0.031**
	0.05	(0.013)	0.221	(0.017)	0.221	0.244	**0.252**
*M*_3_	0.01	(0.002)	0.085	(0.002)	0.085	0.097	**0.103**
	0.001	(0)	0.017	(0)	0.017	0.023	**0.025**

Individual tests results are based on the LRT.

Values in parentheses represent the type 1 error rate.

Highest powers are indicated in bold numbers.

For α_F_ = 0.05, 0.01^*^, 0.001^**^, α = 0.0167, 0.0033^*^, 0.00033^**^, respectively.

**Table 5:** **Power comparisons for sample size 200 based on 1000 replications**.

	**α*_F_***		**Individual tests**				**Simultaneous test**	
		**1**	**2**	**3**	**Union**	**Score**	**LRT**
**STUDY 1**							
	0.05	0.803	0.798	0.375	0.96	**0.983**	**0.983**
*G*_1_	0.01	0.626	0.599	0.192	0.842	0.938	**0.939**
	0.001	0.349	0.326	0.06	0.557	0.772	**0.778**
	0.05	0.783	0.944	0.441	0.984	**0.994**	**0.994**
*G*_2_	0.01	0.588	0.841	0.243	0.927	0.972	**0.974**
	0.001	0.31	0.569	0.086	0.69	**0.888**	**0.888**
	0.05	0.875	0.591	0.301	0.945	0.957	**0.958**
*G*_3_	0.01	0.694	0.366	0.139	0.813	0.895	**0.896**
	0.001	0.394	0.135	0.031	0.476	0.697	**0.704**
	0.05	0.522	0.512	0.209	0.786	0.817	**0.818**
*M*_1_	0.01	0.3	0.29	0.077	0.506	0.646	**0.65**
	0.001	0.115	0.097	0.02	0.208	0.355	**0.365**
	0.05	0.312	0.459	0.162	0.652	0.698	**0.702**
*M*_2_	0.01	0.145	0.239	0.058	0.369	0.459	**0.466**
	0.001	0.035	0.083	0.013	0.117	0.196	**0.205**
	0.05	0.621	0.357	0.182	0.764	0.825	**0.828**
*M*_3_	0.01	0.386	0.179	0.073	0.507	0.613	**0.623**
	0.001	0.162	0.053	0.013	0.213	0.332	**0.339**
**STUDY 2**							
	0.05	0.851	0.676	0.417	0.964	**0.991**	**0.991**
*G*_1_	0.01	0.672	0.447	0.227	0.85	0.946	**0.948**
	0.001	0.417	0.187	0.083	0.552	0.832	**0.841**
	0.05	0.89	(0.02)	0.506	0.927	**0.963**	0.962
*G*_2_	0.01	0.745	(0.004)	0.298	0.81	0.862	**0.869**
	0.001	0.468	(0)	0.1	0.52	0.652	**0.662**
	0.05	(0.009)	0.665	(0.013)	**0.665**	0.659	0.664
*G*_3_	0.01	(0.002)	0.456	(0.001)	**0.456**	0.422	0.431
	0.001	(0)	0.201	(0)	0.201	0.191	**0.204**
	0.05	0.587	0.382	0.225	0.785	0.864	0.864
*M*_1_	0.01	0.372	0.198	0.105	0.531	0.699	**0.704**
	0.001	0.149	0.065	0.025	0.215	0.422	**0.431**
	0.05	0.387	(0.016)	0.19	0.499	0.536	**0.544**
*M*_2_	0.01	0.202	(0.005)	0.064	0.251	0.306	**0.31**
	0.001	0.055	(0)	0.015	0.069	0.089	**0.097**
	0.05	(0.015)	0.441	(0.019)	0.441	0.448	**0.455**
*M*_3_	0.01	(0.001)	0.239	(0.002)	0.239	0.237	**0.246**
	0.001	(0)	0.071	(0.002)	0.071	0.077	**0.083**

Individual tests results are based on the LRT.

Values in parentheses represent the type 1 error rate.

Highest powers are indicated in bold numbers.

For α_F_ = 0.05, 0.01^*^, 0.001^**^, α = 0.0167, 0.0033^*^, 0.00033^**^, respectively.

**Table 6:** **Power comparisons for sample size 300 based on 1000 replications**.

	**α_*F*_**	**Individual tests**				**Simultaneous test**	
		**1**	**2**	**3**	**Union**	**Score**	**LRT**
**STUDY 1**							
	0.05	0.932	0.922	0.58	0.994	**1**	**1**
*G*_1_	0.01	0.83	0.824	0.361	0.963	**0.989**	**0.989**
	0.001	0.615	0.607	0.166	0.828	0.952	**0.953**
	0.05	0.93	0.993	0.648	0.998	**0.999**	**0.999**
*G*_2_	0.01	0.804	0.969	0.448	0.989	**0.996**	**0.996**
	0.001	0.557	0.88	0.22	0.931	**0.988**	**0.988**
	0.05	0.961	0.773	0.475	0.99	**0.994**	**0.994**
*G*_3_	0.01	0.899	0.603	0.272	0.956	**0.982**	**0.982**
	0.001	0.724	0.322	0.091	0.795	0.913	**0.914**
	0.05	0.678	0.683	0.318	0.897	0.933	**0.934**
*M*_1_	0.01	0.471	0.489	0.173	0.737	0.83	**0.832**
	0.001	0.229	0.237	0.054	0.424	**0.617**	0.615
	0.05	0.455	0.653	0.232	0.823	0.859	**0.86**
*M*_2_	0.01	0.242	0.459	0.098	0.603	0.678	**0.683**
	0.001	0.089	0.204	0.024	0.283	0.431	**0.437**
	0.05	0.815	0.531	0.304	0.914	**0.942**	0.94
*M*_3_	0.01	0.638	0.319	0.143	0.764	0.839	**0.842**
	0.001	0.376	0.136	0.04	0.464	**0.616**	**0.616**
**STUDY 2**							
	0.05	0.955	0.829	0.598	0.993	**0.999**	**0.999**
*G*_1_	0.01	0.871	0.657	0.389	0.97	**0.995**	**0.995**
	0.001	0.658	0.411	0.169	0.812	**0.969**	0.968
	0.05	0.982	(0.013)	0.724	0.994	**0.995**	**0.995**
*G*_2_	0.01	0.923	(0.004)	0.512	0.963	**0.984**	**0.984**
	0.001	0.772	(0)	0.251	0.817	0.904	**0.907**
	0.05	(0.016)	0.853	(0.014)	0.853	0.853	**0.857**
*G*_3_	0.01	(0.002)	0.701	(0.003)	**0.701**	0.656	0.662
	0.001	(0)	0.418	(0)	**0.418**	0.357	0.363
	0.05	0.725	0.551	0.351	0.909	0.959	0.96
*M*_1_	0.01	0.524	0.356	0.156	0.715	0.865	**0.869**
	0.001	0.265	0.158	0.057	0.405	0.658	**0.661**
	0.05	0.564	(0.025)	0.263	0.683	0.726	**0.73**
*M*_2_	0.01	0.368	(0.005)	0.11	0.439	0.497	**0.501**
	0.001	0.149	(0)	0.032	0.175	0.246	**0.252**
	0.05	(0.018)	0.638	(0.015)	**0.638**	0.62	0.624
*M*_3_	0.01	(0.003)	0.407	(0.004)	**0.407**	0.38	0.391
	0.001	(0)	0.17	(0)	**0.17**	0.163	0.167

Individual tests results are based on the LRT.

Values in parentheses represent the type 1 error rate.

Highest powers are indicated in bold numbers.

For α_F_ = 0.05, 0.01^*^, 0.001^**^, α = 0.0167, 0.0033^*^, 0.00033^**^, respectively.

### 3.2. Framingham heart study analysis results

In fitting the GLMMs to the four longitudinal traits: log(SBP), log(HDL), LDL, and log(TG), the estimated fixed effects for each confounding covariate, and their associated standard errors (SE) and *p*-values, are presented in Table 7 for each longitudinal trait. The time-invariant covariate sex is strongly significant for all traits with very small asymptotic *p*-values (all ≈ 0). The time-invariant covariate diabetes (diabetes diagnosed at any time during the study, with 0 for no, 1 for yes) is also strongly significant for log(SBP), log(HDL), and log(TG). Their *p*-values are all close to 0. Time-variant covariates bmi and smoking status are significantly associated with four longitudinal traits. Covariates age and alcohol consumed (in ounces/week) are found to have a very significant effect on log(SBP), log(HDL) and log(TG). The number of cigarettes per day has very significant effect on LDL and log(TG). Treatment for lipid (cholrx) significantly reduces the log(SBP), LDL, and log(TD), and treatment for hypertension (htnrx) significantly reduces log(HDL). Note that the R function “bfFixefLMER_F.fnc” is used to select covariates to be included in the GLMM. So, the entry with “−” in Table 7 indicates the exclusion of a covariate in the fitted GLMMs.

**Table 7:** **Fixed effects estimates and their associated standard errors of covariates for each longitudinal trait using GLMMs**.

**Covariates**		**Longitudinal traits**			
	**Coefficients**	**log(SBP)**	**log(HDL)**	**LDL**	**log(TG)**
Sex	Estimate	−0.023	0.266	−3.644	−0.103
	*SE*	0.004	0.01	1.417	0.021
	*p*-value	≈ 0^***^	≈ 0^***^	0.0101	≈ 0^***^
Diabetes	Estimate	0.037	−0.096	–	0.17
	*SE*	0.007	0.016	–	0.034
	*p*-value	≈ 0^***^	≈ 0^***^	–	≈ 0^***^
Age	Estimate	0.002	0.002	–	0.018
	*SE*	0.0001	0.0002	–	0.0005
	*p*-value	≈ 0^***^	≈ 0^***^	–	≈ 0^***^
bmi	Estimate	0.006	−0.015	1.213	0.043
	*SE*	0.0004	0.0007	0.109	0.001
	*p*-value	≈ 0^***^	≈ 0^***^	≈ 0^***^	≈ 0^***^
smk	Estimate	−0.014	−0.01	2.832	0.008
(former)	*SE*	0.004	0.009	1.376	0.021
	*p*-value	0.0019^*^	0.2584	0.0395	0.6965
smk	Estimate	−0.015	−0.086	0.799	0.035
(current)	*SE*	0.004	0.01	1.903	0.03
	*p*-value	0.0013^*^	≈ 0^***^	0.6744	0.2340
alc	Estimate	0.002	0.01	–	0.005
	*SE*	0.0003	0.0006	–	0.001
	*p*-value	≈ 0^***^	≈ 0^***^	–	≈ 0^***^
cigs	Estimate	–	–	0.296	0.002
	*SE*	–	–	0.063	0.001
	*p*-value	–	–	≈ 0^***^	0.0075^*^
cholrx	Estimate	−0.018	–	−37.498	−0.139
	*SE*	0.005	–	1.387	0.022
	*p*-value	0.0006^**^	–	≈ 0^***^	≈ 0^***^
htnrx	Estimate	–	−0.018	–	–
	*SE*	–	−0.007	–	–
	*p*-value	–	0.0087^*^	–	–

**Figure 1:**
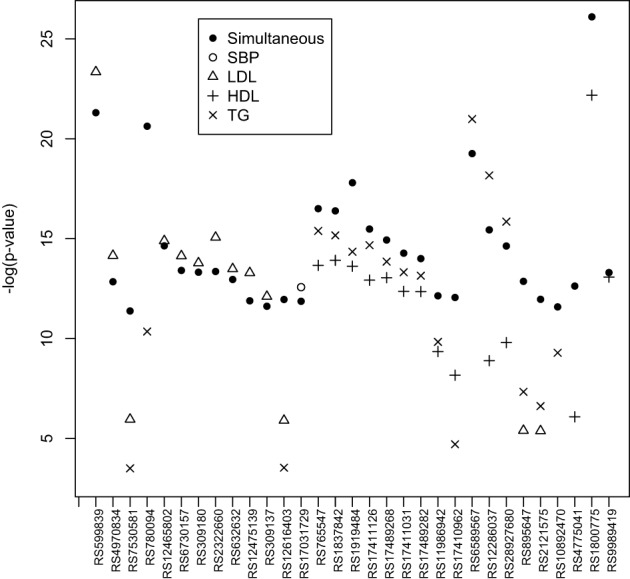
**Most significant SNPs with their −log(*p*-value) from the simultaneous test compared with their significance levels from individual tests**.

In Step 2, we simultaneously test the association between each SNP and all estimated subject-specific effects corresponding to the four traits. We also test the association between each SNP and the estimated subject-specific effect for each trait. SNPs with *p*-value < 1.0 × 10^−5^ from the simultaneous tests (either score test or LRT test) are summarized in Table 8. We also compare their significance levels with those obtained by individual tests in Table 8. Note the *p*-value associated with each individual trait are adjusted via Bonferroni procedure for multiple testing. For easy comparison, results are also presented in Figure 1. In Figure 1, SNPs that are significantly associated with more than one traits generally have a higher −log(*p*-values) or equivalently a lower *p*-value. SNPs that are significantly associated with only one trait have a comparative −log(*p*-value) or equivalently a similar level of significance in *p*-value. On chromosome 8, nine SNPs are found by the simultaneous test to have a strong and significant association with at least one of the four traits. These SNPs are in the *LPL* gene or very close to this gene. The *LPL* gene encodes lipoprotein lipase, a triglyceride hydrolase that acts as a ligand factor for receptor-mediated lipoprotein uptake. According to the individual tests, these nine SNPs are significantly associated with HDL and TG but their *p*-values based on the union of the individual tests are consistently larger than the *p*-values based on the simultaneous test. Note that a larger *p*-value means a less significant level. These significant findings are consistent with other FHS analyses reported by Piccolo et al. (2009) and Ma et al. (2010). SNP RS1800775, less than 0.6 kb from the *CEPT* gene on chromosome 16, is significant in both the simultaneous test (*p*-value = 6.51 × 10^−12^) and the union of the individual tests (*p*-value = 2.32 × 10^−10^). The *CEPT* gene mediates the transfer of cholesterol ester from HDL to other lipoproteins. So, not surprising, this SNP is strongly associated with HDL in the individual test (*p*-value = 5.81 × 10^−11^). This result is also confirmed by Sull et al. (2012) and Sarzynski et al. (2011) in the analyses of independent data sets.

**Table 8:** **Results of most significant SNP (*p*-value < 1.0 × 10^−5^ in simultaneous test)**.

SNP*	**Chr**	**Location (Mb)**	***p*-value**			^**^Associated traits (*p*-value, based on LRT)
			**Score**	**LRT**	**Union**	
RS599839^1−7^	1	109.62	9.35 × 10^−10^	5.59 × 10^−10^	7.24 × 10^−11^	^1−7^LDL(7.24 × 10^−11^)
RS4970834^3−6^	1	109.61	3.21 × 10^−6^	2.64 × 10^−6^	7.21 × 10^−7^	^3,4^LDL(7.12 × 10^−7^)
RS7530581	1	161.11	9.62 × 10^−6^	1.14 × 10^−5^	2.58 × 10^−3^	LDL(2.58 × 10^−3^)
						TG(3.0 × 10^−2^)
RS780094^5,8^	2	27.59	1.30 × 10^−9^	1.10 × 10^−9^	3.19 × 10^−5^	^5,8^TG(3.19 × 10^−5^)
RS12465802^1^	2	136.1	4.73 × 10^−7^	4.38 × 10^−7^	3.40 × 10^−7^	^1^LDL(3.4 × 10^−7^)
RS6730157^1^	2	135.62	1.60 × 10^−6^	1.50 × 10^−6^	7.24 × 10^−7^	^1^LDL(7.24 × 10^−7^)
RS309180^1^	2	136.33	1.64 × 10^−6^	1.64 × 10^−6^	1.03 × 10^−6^	^1^LDL(1.03 × 10^−6^)
RS2322660^1^	2	136.27	1.58 × 10^−6^	1.58 × 10^−6^	2.87 × 10^−7^	^1^LDL(2.87 × 10^−6^)
RS632632^1^	2	136.35	2.37 × 10^−6^	2.36 × 10^−6^	1.38 × 10^−6^	^1^LDL(1.38 × 10^−6^)
RS12475139	2	136.50	7.02 × 10^−6^	6.86 × 10^−6^	1.68 × 10^−6^	LDL(1.68 × 10^−6^)
RS309137	2	136.48	9.03 × 10^−6^	9.01 × 10^−6^	5.52 × 10^−6^	LDL(5.52 × 10^−6^)
RS12616403	2	85.13	6.67 × 10^−6^	6.42 × 10^−6^	2.73 × 10^−3^	LDL(2.73 × 10^−3^)
						TG(2.90 × 10^−2^)
RS17031729	3	63.39	7.89 × 10^−6^	7.03 × 10^−6^	3.46 × 10^−6^	SBP(3.46 × 10^−6^)
RS765547^1,3^	8	19.91	9.52 × 10^−8^	6.81 × 10^−8^	2.08 × 10^−7^	^3^HDL(1.16 × 10^−6^)
						^3^TG(2.08 × 10^−7^)
RS1837842^1,3^	8	19.91	1.05 × 10^−7^	7.62 × 10^−8^	2.588 × 10^−7^	^3^HDL(9.04 × 10^−7^)
						^3^TG(2.588 × 10^−8^)
RS1919484^1,3,9^	8	19.91	2.50 × 10^−7^	1.86 × 10^−8^	5.89 × 10^−7^	^3,9,1^HDL(1.21 × 10^−6^)
						^3^TG(5.89 × 10^−7^)
RS17411126^1,3^	8	19.9	2.54 × 10^−7^	1.89 × 10^−7^	4.24 × 10^−7^	^3,7^HDL(2.44 × 10^−6^)
						^3^TG(4.24 × 10^−7^)
RS17489268^1,3^	8	19.9	4.37 × 10^−7^	3.27 × 10^−7^	9.69 × 10^−7^	^3^HDL(2.16 × 10^−6^)
						^3^TG(9.69 × 10^−7^)
RS17411031^1,3,5^	8	19.9	8.33 × 10^−7^	6.33 × 10^−7^	1.64 × 10^−6^	^1,3,5^HDL(4.28 × 10^−6^)
						^3^TG(1.64 × 10^−6^)
RS17489282^1^	8	19.9	1.02 × 10^−6^	8.30 × 10^−7^	1.95 × 10^−6^	HDL(4.32 × 10^−6^)
						TG(1.95 × 10^−6^)
RS11986942^1,3^	8	19.91	6.87 × 10^−6^	5.34 × 10^−6^	5.37 × 10^−5^	^3^HDL(8.68 × 10^−5^)
						TG(5.37 × 10^−5^)
RS17410962^1,3^	8	19.89	9.60 × 10^−6^	5.80 × 10^−6^	2.84 × 10^−4^	^3^HDL(2.84 × 10^−4^)
						TG(9 × 10^−3^)
RS6589567^10^	11	116.18	3.54 × 10^−9^	4.33 × 10^−9^	7.66 × 10^−10^	^10^TG(7.66 × 10^−10^)
RS12286037^7,8,11^	11	116.16	1.59 × 10^−7^	1.97 × 10^−7^	1.29 × 10^−8^	HDL(1.373 × 10^−4^)
						^7^TG(1.29 × 10^−8^)
RS28927680^8,11−13^	11	116.12	4.46 × 10^−7^	4.42 × 10^−7^	1.29 × 10^−7^	^12,13^HDL(5.56 × 10^−5^)
						^8,12^TG(1.29 × 10^−7^)
RS895647	11	119.19	2.83 × 10^−6^	2.59 × 10^−6^	6.53 × 10^−4^	LDL(4.52 × 10^−3^)
						TG(6.53 × 10^−4^)
RS2121575	11	119.18	6.90 × 10^−6^	6.37 × 10^−6^	1.33 × 10^−3^	LDL(4.6 × 10^−3^)
						TG(1.33 × 10^−3^)
RS10892470	11	119.18	1.07 × 10^−5^	9.30 × 10^−6^	9.26 × 10^−5^	TG(9.26 × 10^−5^)
RS4775041	15	56.46	3.65 × 10^−6^	3.28 × 10^−6^	2.28 × 10^−3^	HDL(2.28 × 10^−3^)
RS1800775^1,11−15^	16	55.55	6.51 × 10^−12^	4.62 × 10^−12^	2.32 × 10^−10^	^1,13−15^HDL(2.32 × 10^−10^)
RS9989419^1,3,5,13,15^	16	55.54	1.80 × 10^−6^	1.67 × 10^−6^	2.09 × 10^−6^	^1,3,5,13,15^HDL(2.09 × 10^−6^)

^*^ and ^**^: Literature confirmation by simultaneous test and individual tests, respectively.

1 , Ma et al., 2010;

2, Roslin et al., 2009;

3 , Piccolo et al., 2009;

4 , Muendlein et al., 2009;

5 , Wallace et al., 2008;

6 , Suchindran et al., 2010;

7 , Mohlke et al., 2008;

8 , Hegele et al., 2009;

9 , Chen et al., 2012;

10 , Clark et al., 2012;

11 , Sabatti et al., 2009;

12 , Hamid et al., 2009;

13 , Boes et al., 2009;

14 , Sull et al., 2012;

15 , Sarzynski et al., 2011.

On chromosome 11, 3 SNPs (RS6589567, RS12286037, and RS28927680) are found to be significantly associated with HDL and/or TG traits. These SNPs are either in or close to the *APOA5* gene which is known to play an important role in regulating TG level and is a component of HDL. This gene is also known as a major risk factor for coronary artery disease and is associated with hypertriglyceridemia, and hyperlipoproteinemia type 3. These significant findings are also reported by others (Mohlke et al., 2008; Boes et al., 2009; Hamid et al., 2009; Hegele et al., 2009; Sabatti et al., 2009; Clark et al., 2012). About 3Mb away from these SNPs, three other SNPs (RS895647, RS2121575, and RS10892470) are significantly associated with LDL and/or TG. These SNPs are very close to the *POU2F3* gene which is known to associate with coronary thrombosis. On chromosome 2, we also find that SNP RS12616403 is significantly associated with LDL and TG. This SNP is in the *KCMF1* (potassium channel modulatory factor 1) gene which is known to associate with maturity-onset diabetes of the young.

## 4. Discussion

In this paper, we proposed a two-step procedure for a genetic association analysis with multiple longitudinal traits. In the first step, a GLMM is used to analyze each longitudinal trait individually. This allows us to flexibly incorporate different covariate sets that are relevant to different longitudinal traits and also to flexibly handle traits of different data types. In the GLMMs, unmeasured subject-specific genetic effects are packed into the random effects term while accounting for the fixed effects of other confounding factors. With a longitudinal study design, repeated measurements on each subject enable the estimation of subject-specific effects. This has been validated by our simulation study that included genetic effects. With the 2-step approach, the method has the advantage of being able to efficiently and simultaneously test a large-scale genome-wide SNP associations with multiple traits in the second step, with the fixed effects of the potential confounding factors for each trait taken into account in the first step. Then, subsequent individual tests would be performed on a much smaller subset of significant SNPs found by this two-step procedure to further investigate which particular traits are associated with the SNPs.

Our proposed method opens several avenues for future research. For example, a specific gene-environmental interaction can be modeled by the introduction of a random slope term in the GLMM. However, there are a small number of repeated measurements and many possible gene-interacting environmental factors. Therefore, it is worthwhile to investigate an efficient procedure to incorporate gene-environmental interaction terms in the GLMMs, and perform genome-wide association tests for these interactions. The proposed method is for a single marker test only. When there are gene-gene interactions, but only one of the markers is tested marginally, the power to detect genetic association may be comprised. Moreover, our proposed method is a logistic regression method. It is reported that the probability of a rare event can be underestimated by logistic regression (King and Zeng, 2001). So, when testing a rare variant, the minor allele frequency of the variant can be underestimated, and the test statistic may not follow the expected asymptotic distribution. Therefore, it is worthwhile to further investigate a robust logistic regression method for testing on rare as well as common variants.

Our current method focuses only on the analysis of unrelated individuals, so possible future research would be to extend the current method to the analysis of family data. When family data are analyzed, a three-level nested mixed effects model can be used in which repeated measurements (level 1) are nested within subjects (level 2) and subjects are nested within families (level 3). When testing the association between a SNP and subject-specific effects, the response in the binomial regression model is the allele frequency of the SNP, so observed responses are no longer independent due to the relationship among related subjects. The current score test and likelihood ratio test based on the independent subjects assumption would no longer be applicable. A modified method such as the quasi-likelihood based method could be considered.

In reality, the random effects γ_*ij*_ s are not observable. In the analysis, we replace the true random effect γ_*ij*_ by the estimated random effect, $\hat{\gamma }$_*ij*_, obtained in the first step. Since the random effect is treated as a covariate in the second step, issues of measurement errors may be of concern. Based on a Taylor expansion, Rosner et al. (1990) proposed a first order approximation to derive a corrected estimate, and Kuha (1994) derived a corrected estimate based on the second order approximation. We applied first-order and second-order approximations to corrected estimates of random effects. The first order correction gives an identical estimate γ_*ij*_ as we obtained from Step 1. The second-order correction leads to a more complicated estimator with higher computation cost. However, results from simulation studies show that using the second order corrected estimate only improves the power slightly, about 0.1%, in several settings. For this reason, we did not pursue the measurement error correction further in our paper.

Finally, it is worth mentioning the missing data problem that commonly occurs in longitudinal studies. In general, there are three missing data scenarios in longitudinal data. For example, in the FHS, some participants missed a particular examination such that all measurements at that particular time point are missing. In other situations, some subjects participated at an examination but the information on the measurements at that time point is somehow incomplete. The last missing data scenario would be when some subjects dropout from the study and thus measurements are discontinued. Under the assumption of a missing completely at random (MCAR) mechanism, our method is applicable for subjects that have different numbers of measurements. However, when the MCAR assumption is invalid, methods of handling missing data under a different mechanism, such as missing not at random (MNAR), should be considered in order to obtain unbiased estimates for fixed effects of covariates as well as the subject-specific random effects.

## Author contributions

Weiqiang Wang and Zeny Feng developed and implemented the method, and performed simulation studies and FHS data analysis. Zeny Feng supervised the project. Shelley B. Bull and Zuoheng Wang provided constructive comments and suggestions. All authors drafted, read and approved the final manuscript.

### Conflict of interest statement

The authors declare that the research was conducted in the absence of any commercial or financial relationships that could be construed as a potential conflict of interest.
