# Supplementary material for: A 2-step strategy for detecting pleiotropic effects on multiple longitudinal traits
Source: Front Genet. 2014 Oct 20;5:357. doi: 10.3389/fgene.2014.00357 (PMC4202779; doi:10.3389/fgene.2014.00357)
Supplement: Supplementary file 1 [file Presentation1.PDF]

Supplementary Materials for “A 2-step strategy for detecting pleiotropic effects on multiple longitudinal traits”

Weiqiang Wang [1], Zeny Feng [1,*], Shelley Bull [2] and Zuoheng Wang [3]

[1] *Department of Mathematics and Statistics, University of Guelph, Guelph, Ontario, Canada*
[2] *Lunenfeld-Tanenbaum Research Institute of Mount Sinai Hospital, Prosserman Centre for Health Research, and Dalla Lana School of Public Health, University of Toronto, Toronto, Ontario, Canada*
[3] *Division of Biostatistics, Yale School of Public Health, New Haven, Connecticut, USA.*

Correspondence*:
Zeny Feng
Department of Mathematics and Statistics, University of Guelph, Guelph, Ontario, N1G 2W1, Canada, zfeng@uoguelph.ca

1 ADDITIONAL SIMULATION STUDIES AND RESULTS

The settings of two additional simulation studies, denoted as Study A1 and Study A2, are very similar to the settings of the two studies in the main paper. The similarity of the settings include: the number of traits, the data type of each trait, the number of causal SNPs, and the number of SNPs affecting each trait (see Table 1), allele frequencies of the three causal SNPs and the three associated SNPs, the effects of each causal SNP to each trait, and allele frequency of the SNP M that is not associated with any trait in both studies. However, in the additional studies, we consider a covariate of count data type in the simulation model. Consider the linear predictor as shown in the main paper is given by

$$\eta_{ijt} = g(\mu_{ijt}) = \alpha_{j0} + \alpha_{j1}Z_{it1} + \alpha_{j2}Z_{it2} + b_{j1}G_{i1} + b_{j2}G_{i2} + b_{j3}G_{i3},$$

for $i = 1, ..., n, j = 1, 2, 3,$ and $t = 1, ..., 5$. Again, Z_{it1}'s are generated from $N(40, 7^2)$ to micmic the age distribution of patients. For Z_{it2}, we let 30% of subjects have $Z_{it2} = 0$ for all t and the rest of 70% of subjects with Z_{it2}'s being generated from a Poisson distribution with mean of 5. This is to micmic the number of cigarette consumed by each subject every day. We set $\alpha_1 = (0, 0.4, 0.3)$ for the first trait, $\alpha_2 = (0, 0.4, -0.2)$ for the second trait, and $\alpha_3 = (-0.4, -0.5, 0.4)$ for the third trait.

For each simulation study, we generate samples of size $n = 100, 200,$ and 300 and, for each specified sample size, we simulate 1000 data sets. For each data set, we first fit the GLMM to obtain an estimate of γ_{ij} for each trait and each subject. In the GLMMs, both covariates, Z_{it1} and Z_{it2}, are included. For each SNP, we then perform a simultaneous test on all three estimated subject-specific effects; $\hat{\boldsymbol{\gamma}}_1$, $\hat{\boldsymbol{\gamma}}_2$ and $\hat{\boldsymbol{\gamma}}_3$, where each $\hat{\boldsymbol{\gamma}}_j = (\hat{\gamma}_{1j}, ..., \hat{\gamma}_{nj})^T$ is treated as a phenotype. Because we simultaneously test on three phenotypes, both W_s and Λ test statistics follow a χ^2_3 distribution asymptotically under the null hypothesis. We reject the null hypothesis if the test statistic is greater than the $(1 - \alpha_F)$th quantile of the χ^2_3 distribution. We let $\alpha_F = 0.05, 0.01,$ and 0.001. We also perform individual association tests between each SNP and each subject-specific effect for each trait. We reject the null hypothesis if the test statistic computed for only one estimated subjected-specific effect has a value greater than the $(1 - \alpha)$th quantile of χ^2_1 distribution. Here, α is given by $\alpha_F = 1 - (1 - \alpha)^3$ and α_F is the family-wise error rate (FWER) controlling at 0.05, 0.01, and 0.001 levels.

Table 1. SNP effects on three traits for simulation Study A1 and A2

Study A1				Study A2			
	Trait				Trait		
SNP	X_1	X_2	X_3	SNP	X_1	X_2	X_3
G_1	Yes	Yes	Yes	G_1	Yes	Yes	Yes
G_2	Yes	Yes	Yes	G_2	Yes	No	Yes
G_3	Yes	Yes	Yes	G_3	No	Yes	No

Table 2. Mean and standard error of fixed effects estimates using GLMMs and based on over 1000 simulations of sample size 100.

		Study A1		Study A2	
Traits	Fixed Effect	Estimate	SE	Estimate	SE
1	$\alpha_{11} = 0.4$	0.400439	0.006622	0.399392	0.006436
	$\alpha_{12} = 0.3$	0.300109	0.01553	0.298747	0.015557
2	$\alpha_{21} = 0.4$	0.399784	0.006369	0.40017	0.006346
	$\alpha_{22} = -0.2$	-0.20103	0.016252	-0.20014	0.015239
3	$\alpha_{31} = -0.5$	-0.52875	0.059557	-0.52836	0.060522
	$\alpha_{32} = 0.4$	0.420952	0.071897	0.422385	0.071392

In Table 2, the mean and standard error of fixed effects estimated, $\hat{\alpha}$'s over 1000 simulation on sample size of 100 are reported and compared with the true values of each fixed effect. The results of both studies show that the GLMMs generally give unbiased estimates for the fixed effect parameters with small standard errors.

For SNP M that is not associated with any trait, the empirical null rejection rates for either studies and for the combined studies (indicated as "Study A1+A2") are reported in Table 3. For individual tests, we only report the results based on the likelihood ratio test (LRT). The result indicated the empirical null rejection rate are very close to their corresponding nominal levels.

The empirical power for each causal SNP and associated SNP are reported in Table 4-6. Again, similar pattern in terms of power comparison between the simultaneous test and the union of individual tests is obtained. When a SNP affects more than one trait, the simultaneous test is generally more powerful than the union of individual tests. When a SNP affects only one trait, the power between simultaneous test and individual test is comparative.

Table 3. Type I error rate assessment based on 1000 simulations in each study.

Sample size		Individual tests				Simultaneous test	
$n = 100$	α_F	1	2	3	union	score	LRT
Study A1	0.05	0.013	0.015	0.011	0.039	0.048	0.05
	0.01	0.003	0.003	0.002	0.008	0.009	0.010
	0.001	0	0.001	0	0.001	0	0
Study A2	0.05	0.017	0.018	0.018	0.048	0.046	0.049
	0.01	0.008	0.001	0.003	0.012	0.014	0.016
	0.001	0	0	0	0	0	0.001
Study A1+A2	0.05	0.015	0.0165	0.0145	0.0435	0.047	0.0495
	0.01	0.0055	0.002	0.0025	0.01	0.0115	0.013
	0.001	0	0.0005	0	0.0005	0	0.0005
$n = 200$							
Study A1	0.05	0.014	0.017	0.014	0.042	0.042	0.044
	0.01	0.001	0.003	0.003	0.007	0.014	0.014
	0.001	0	0	0	0.001	0	0
Study A2	0.05	0.018	0.012	0.013	0.049	0.042	0.049
	0.01	0.002	0.005	0.003	0.01	0.005	0.009
	0.001	0	0	0.001	0.001	0	0.001
Study A1+A2	0.05	0.016	0.0145	0.0115	0.0455	0.042	0.0465
	0.01	0.0015	0.004	0.003	0.0085	0.0095	0.0115
	0.001	0	0	0.0005	0.001	0	0.0005
$n = 300$							
Study A1	0.05	0.006	0.018	0.017	0.041	0.048	0.048
	0.01	0	0.005	0.004	0.009	0.01	0.012
	0.001	0.001	0	0	0.001	0	0
Study A2	0.05	0.014	0.022	0.017	0.051	0.052	0.053
	0.01	0.002	0.003	0.001	0.006	0.007	0.008
	0.001	0.001	0	0	0.001	0	0.001
Study 1+2	0.05	0.01	0.02	0.017	0.046	0.05	0.0505
	0.01	0.001	0.004	0.0025	0.0075	0.085	0.01
	0.001	0.001	0	0	0.001	0	0.0005

Individual test results are based on the LRT.
For $\alpha_F = 0.05, 0.01, 0.001, \alpha = 0.0167, 0.0033, 0.00033$, respectively.

Table 4. Power comparisons for sample size 100 based on 1000 replications.

Study A1	α_F	Individual tests 1	2	3	union	Simultaneous test score	LRT
G_1	0.05	0.377	0.393	0.111	0.623	0.704	**0.713**
	0.01	0.194	0.204	0.033	0.348	0.467	**0.487**
	0.001	0.046	0.051	0.004	0.092	0.217	**0.237**
G_2	0.05	0.358	0.557	0.142	0.716	0.789	**0.793**
	0.01	0.174	0.34	0.046	0.462	0.572	**0.587**
	0.001	0.043	0.128	0.007	0.268	0.316	**0.334**
G_3	0.05	0.495	0.244	0.102	0.63	0.6777	**0.683**
	0.01	0.267	0.109	0.025	0.346	0.425	**0.444**
	0.001	0.074	0.032	0.005	0.103	0.187	**0.202**
M_1	0.05	0.221	0.217	0.069	0.405	0.453	**0.46**
	0.01	0.1	0.086	0.012	0.178	**0.24**	0.237
	0.001	0.024	0.018	0.002	0.041	0.083	**0.09**
M_2	0.05	0.126	0.208	0.056	0.329	0.367	**0.372**
	0.01	0.047	0.083	0.011	0.133	0.164	**0.179**
	0.001	0.006	0.019	0.001	0.026	0.048	**0.058**
M_3	0.05	0.291	0.152	0.066	0.402	0.45	**0.457**
	0.01	0.119	0.047	0.015	0.167	0.23	**0.246**
	0.001	0.029	0.011	0.002	0.039	0.068	**0.079**
Study A2							
G_1	0.05	0.426	0.282	0.115	0.624	0.739	**0.754**
	0.01	0.221	0.114	0.035	0.323	0.527	**0.549**
	0.001	0.055	0.025	0.006	0.081	0.235	**0.263**
G_2	0.05	0.485	(0.013)	0.147	0.56	0.619	**0.624**
	0.01	0.268	(0.002)	0.06	0.312	0.364	**0.389**
	0.001	0.088	(0)	0.009	0.096	0.152	**0.17**
G_3	0.05	(0.013)	0.292	(0.015)	0.292	0.322	**0.333**
	0.01	(0.02)	0.123	(0.001)	0.123	0.133	**0.144**
	0.001	(0)	0.023	(0)	0.023	0.034	**0.04**
M_1	0.05	0.227	0.164	0.066	0.379	0.472	**0.489**
	0.01	0.087	0.066	0.013	0.153	0.23	**0.243**
	0.001	0.027	0.011	0.002	0.039	0.085	**0.093**
M_2	0.05	0.166	(0.014)	0.071	0.223	0.255	**0.265**
	0.01	0.056	(0.004)	0.018	0.073	0.091	**0.107**
	0.001	0.01	(0)	0.002	0.0119	0.025	**0.031**
M_3	0.05	(0.015)	0.168	(0.011)	0.168	0.209	**0.219**
	0.01	(0.002)	0.063	(0.002)	0.063	0.072	**0.081**
	0.001	(0)	0.011	(0)	0.011	0.012	**0.016**

Individual tests results are based on the LRT.
Values in parentheses represent the type 1 error rate.
Highest powers are indicated in bold numbers.
For $\alpha_F = 0.05, 0.01, 0.001, \alpha = 0.0167, 0.0033, 0.00033$, respectively.

Table 5. Power comparisons for sample size 200 based on 1000 replications.

	α_F	Individual tests				Simultaneous test	
Study A1		1	2	3	union	score	LRT
G_1	0.05	0.76	0.764	0.286	0.942	**0.969**	**0.969**
	0.01	0.571	0.551	0.114	0.795	0.899	**0.896**
	0.001	0.292	0.272	0.02	0.47	0.702	**0.707**
G_2	0.05	0.707	0.913	0.299	0.973	**0.992**	**0.992**
	0.01	0.483	0.787	0.149	0.88	0.941	**0.943**
	0.001	0.226	0.529	0.029	0.617	0.803	**0.811**
G_3	0.05	0.824	0.526	0.223	0.912	0.934	**0.938**
	0.01	0.645	0.311	0.09	0.759	0.83	**0.835**
	0.001	0.388	0.124	0.016	0.461	0.635	**0.64**
M_1	0.05	0.504	0.455	0.151	0.736	0.781	**0.785**
	0.01	0.278	0.25	0.065	0.47	0.592	**0.594**
	0.001	0.092	0.094	0.011	0.184	0.32	**0.325**
M_2	0.05	0.272	0.423	0.12	0.601	0.661	**0.663**
	0.01	0.131	0.231	0.036	0.334	0.405	**0.409**
	0.001	0.034	0.074	0.002	0.107	0.189	**0.199**
M_3	0.05	0.604	0.314	0.137	0.728	0.763	**0.765**
	0.01	0.363	0.142	0.054	0.46	0.562	**0.565**
	0.001	0.148	0.043	0.009	0.182	0.278	**0.295**
Study A2							
G_1	0.05	0.782	0.566	0.286	0.9432	0.925	**0.976**
	0.01	0.565	0.348	0.12	0.75	0.731	**0.897**
	0.001	0.3	0.13	0.033	0.411	0.412	**0.709**
G_2	0.05	0.849	(0.026)	0.397	0.908	**0.965**	0.923
	0.01	0.648	(0.001)	0.191	0.715	**0.815**	0.787
	0.001	0.357	(0)	0.05	0.389	0.487	**0.536**
G_3	0.05	(0.016)	0.621	(0.01 0)	**0.621**	**0.621**	0.605
	0.01	(0.001)	0.376	(0.004)	0.376	**0.377**	0.354
	0.001	(0)	0.153	(0)	0.153	**0.154**	0.135
M_1	0.05	0.487	0.312	0.138	0.695	0.671	**0.765**
	0.01	0.259	0.141	0.049	0.394	0.38	**0.56**
	0.001	0.094	0.039	0.01	0.138	0.131	**0.3**
M_2	0.05	0.361	(0.02)	0.106	0.428	0.464	**0.477**
	0.01	0.171	(0.005)	0.044	0.207	**0.259**	0.244
	0.001	0.045	(0.001)	0.011	0.055	**0.109**	0.08
M_3	0.05	(0.011)	0.385	(0.019)	0.385	0.385	**0.388**
	0.01	(0.002)	0.185	(0.002)	0.185	**0.186**	0.177
	0.001	(0)	0.055	(0)	0.055	**0.056**	0.046

Individual tests results are based on the LRT.
Values in parentheses represent the type 1 error rate.
Highest powers are indicated in bold numbers.
For $\alpha_F = 0.05, 0.01, 0.001$, $\alpha = 0.0167, 0.0033, 0.00033$, respectively.

Table 6. Power comparisons for sample size 300 based on 1000 replications.

Study A1	α_F	Individual tests 1	2	3	union	Simultaneous test score	LRT
G_1	0.05	0.913	0.92	0.408	0.99	**0.995**	**0.995**
	0.01	0.808	0.791	0.216	0.952	**0.981**	**0.981**
	0.001	0.573	0.547	0.074	0.802	0.941	**0.944**
G_2	0.05	0.707	0.913	0.299	0.973	**0.992**	**0.992**
	0.01	0.881	0.985	0.468	0.998	**1**	**1**
	0.001	0.73	0.949	0.274	0.986	**0.995**	**0.995**
G_3	0.05	0.949	0.751	0.294	0.982	**0.993**	**0.993**
	0.01	0.875	0.542	0.142	0.931	0.968	**0.969**
	0.001	0.646	0.273	0.041	0.726	0.875	**0.881**
M_1	0.05	0.674	0.652	0.201	0.895	**0.921**	**0.921**
	0.01	0.45	0.447	0.096	0.687	0.799	**0.803**
	0.001	0.218	0.209	0.015	0.374	0.543	**0.549**
M_2	0.05	0.399	0.634	0.143	0.79	0.832	**0.835**
	0.01	0.207	0.409	0.052	0.539	0.648	**0.653**
	0.001	0.068	0.169	0.01	0.226	0.362	**0.365**
M_3	0.05	0.776	0.513	0.178	0.887	**0.922**	**0.922**
	0.01	0.582	0.3	0.072	0.694	0.781	**0.783**
	0.001	0.325	0.114	0.02	0.397	0.551	**0.559**
Study A2							
G_1	0.05	0.94	0.794	0.396	0.99	**1**	**1**
	0.01	0.832	0.596	0.208	0.934	**0.99**	**0.99**
	0.001	0.591	0.313	0.066	0.716	0.926	**0.934**
G_2	0.05	0.954	(0.025)	0.534	**0.993**	0.982	0.982
	0.01	0.868	(0.004)	0.292	**0.954**	0.94	0.943
	0.001	0.698	(0.001)	0.112	0.804	0.822	**0.83**
G_3	0.05	(0.021)	0.784	(0.013)	**0.784**	0.777	0.778
	0.01	(0.004)	0.594	(0.001)	**0.594**	0.55	0.556
	0.001	(0)	0.312	(0)	**0.313**	0.268	0.275
M_1	0.05	0.703	0.492	0.223	0.872	0.928	**0.93**
	0.01	0.482	0.269	0.094	0.643	0.791	**0.796**
	0.001	0.228	0.118	0.023	0.325	0.539	**0.549**
M_2	0.05	0.524	(0.012)	0.187	0.609	0.643	**0.648**
	0.01	0.309	(0.003)	0.063	0.368	0.416	**0.423**
	0.001	0.103	(0.001)	0.011	0.113	0.149	**0.163**
M_3	0.05	(0.013)	0.528	(0.013)	0.528	0.532	**0.536**
	0.01	(0.001)	0.347	(0.001)	**0.347**	0.319	0.321
	0.001	(0)	0.139	(0)	**0.139**	0.111	0.115

Individual tests results are based on the LRT.
Values in parentheses represent the type 1 error rate.
Highest powers are indicated in bold numbers.
For $\alpha_F = 0.05, 0.01, 0.001$, $\alpha = 0.0167, 0.0033, 0.00033$, respectively.
